# Assessment of Risk Factors for Cognitive Impairment Accounting for Genetic and Environmental Influences: An Italian Population-Based Twin Study

**DOI:** 10.3390/brainsci15111197

**Published:** 2025-11-07

**Authors:** Emanuela Medda, Nicola Vanacore, Marco Canevelli, Francesco Sciancalepore, Elisa Fabrizi, Nicoletta Locuratolo, Filippo Nuti, Corrado Fagnani

**Affiliations:** 1 Italian Twin Registry, Centre for Behavioural Sciences and Mental Health, Italian National Institute of Health, 00161 Rome, Italy; corrado.fagnani@iss.it; 2 National Centre for Disease Prevention and Health Promotion, Italian National Institute of Health, 00161 Rome, Italy; nicola.vanacore@iss.it (N.V.); francesco.sciancalepore@iss.it (F.S.); elisa.fabrizi@iss.it (E.F.); nicoletta.locuratolo@iss.it (N.L.); 3 Department of Human Neuroscience, Sapienza University of Rome, 00185 Rome, Italy; marco.canevelli@gmail.com (M.C.); filippo.nuti@uniroma1.it (F.N.)

**Keywords:** twins, dementia, genetics, environment, risk factors, cognition

## Abstract

**Background/Objectives:** The etiology of dementia is complex and multifactorial, with both genetic and environmental factors contributing to its onset. The Lancet Commission has identified several risk factors for this condition, but it is increasingly urgent to confirm their etiological role while accounting for both measured and unmeasured confounding effects. Our study, conducted on a population-based sample of Italian twins, examines the link between known risk factors and cognitive impairment, and the contribution of genetic and environmental influences to this link. **Methods:** Study participants were adult twins from the Italian Twin Registry who completed self-administered questionnaires. Cognitive impairment was evaluated by the SAGE questionnaire, while risk factors were assessed using the checklist proposed by the Lancet Commission. Individual-level and matched-pair analyses were performed for each risk factor, and their results were compared to detect potential genetic or environmental confounding, and to infer “quasi-causality” in the examined associations. **Results:** A total of 483 twins participated in the study (mean age 69.14 years, 63% women, 47% monozygotic twins). In matched-pair analyses, the association between hearing loss and cognitive impairment decreased in magnitude and became non-significant, suggesting confounding by genetics or early-life environment; in contrast, the association with sleep disturbances resulted strong and significant in both individual-level and matched-pair analyses, indicating a genuine effect of sleep on cognition. **Conclusions:** Our findings suggest a potential “quasi-causal” role of sleep disorders in cognitive decline. This relationship should be clarified through well-powered longitudinal studies incorporating precise clinical definitions and biomarker data.

## 1. Introduction

The Lancet Commission on dementia prevention, intervention, and care 2024 estimated that up to 45% of dementia cases could be preventable by targeting 14 risk factors [1]. In the context of public health, there is ongoing discussion regarding the evidence available on whether it is more effective to implement dementia prevention interventions at the individual or population levels [2]. Recently, several policy documents in England have emerged, focusing on primary prevention strategies aimed at individuals and populations. Additionally, favourable economic models have been proposed to support a population-based approach to dementia prevention [3,4].

Simultaneously, discussions are taking place regarding how observational studies in dementia prevention can establish causal links between exposures and outcomes [1,5]. Although randomized controlled trials (RCTs) are regarded as the gold standard for assessing intervention efficacy, they are often impractical for studying dementia prevention due to issues related to feasibility, participant vulnerability, and informed consent. In contrast, observational studies that focus on dementia risk factors face limitations related to confounding, reverse causation, and selection biases. Various approaches have been proposed to assess causality in dementia prevention, including Mendelian randomization analyses [6], quasi-experimental studies [5], and the target trial framework in observational study [7]. Moreover, a life course epidemiology approach may allow to study the effect of possible risk factors on dementia across the lifespan from a social, psychological, behavioural, and biological perspective [8]. From a methodological standpoint, in observational studies that evaluate multiple risk factors in relation to the onset of complex diseases, adjusting for potential confounders for each risk factor separately has recently been recommended [9]. Ultimately, the etiology of dementia is complex and multifactorial, encompassing contributions from genetic, epigenetic, lifestyle, and environmental factors [10]. This complexity underscores the urgency of evaluating the relationship between multimorbidity and environmental and genetic factors in the onset of cognitive disorders with appropriate study designs [11].

In this context, the co-twin control study may serve as an effective study design for controlling a broad range of confounders, both genetic and environmental, and then to investigate the causal relation between multiple exposure factors and a given outcome [12,13,14]. This approach has previously been applied only to a subset of modifiable risk factors associated with the onset of dementia, including cardiovascular diseases, traumatic brain injuries (TBI) and alcohol consumption. For instance, an analysis involving 272 male monozygotic and dizygotic twin pairs from the Vietnam Era Twin Registry revealed that the association between brain health and cardiovascular health was largely attributable to familial factors, indicating the influence of early life exposures [15]. Similarly, a subsequent co-twin design study conducted with 356 monozygotic and dizygotic twin pairs from the Swedish Twin Registry found that the association between cardiometabolic diseases and dementia was partly explained by genetic factors shared by both conditions [16]. Additionally, co-twin controlled analyses involving 1195 monozygotic and dizygotic twin pairs discordant for TBI from the National Academy of Sciences-National Research Council’s Twin Registry of male World War II veterans suggested a causal relationship between TBI and poorer cognitive outcomes [17]. Furthermore, an analysis of 576 discordant monozygotic and dizygotic twin pairs from the Swedish Twin Registry indicated that moderate to heavy drinking among twins was associated with an increased risk of dementia, suggesting that genetic and/or familial factors do not account for these associations [18]. For all the other known risk factors of cognitive decline and dementia, no previous studies in non-clinical samples have used a genetically informed design—like the co-twin control approach—to control for genetic and early environmental confounding effects in the observed associations. Therefore, we aimed to fill this gap considering a general population sample of twins from the Italian Twin Registry (ITR).

The ITR was established in 2001 at the Istituto Superiore di Sanità (Italian National Institute of Health, ISS) as a population-based registry comprising volunteer twins [19]. Upon enrolment, twins consent to participate in the registry and to be recontacted for future research initiatives.

Thus, twins offer a distinctive opportunity to explore the role of familial influences in the etiology of cognitive disorders. The objectives of the present study are as follows: (1) to investigate the overall association of known risk factors for cognitive decline and dementia with potential cognitive impairment in a non-clinical sample of Italian adult twins and (2) to evaluate if this association may be explained by genetic and environmental confounding factors, by using a matched design based on monozygotic and dizygotic twin pairs.

## 2. Materials and Methods

### 2.1. Study Design and Participants

Participants were adult twins previously enrolled in the ITR who agreed to take part in the study upon recontact. The registry includes pairs of monozygotic (MZ) and dizygotic (DZ) twins, who share 100% and approximately 50% of their genetic backgrounds, respectively; furthermore, both types of twins share early-life environmental factors, including family exposures and the intrauterine environment.

Data collection was carried out using self-administered questionnaires, which were mailed to participants’ homes, and once completed, returned to the ITR via postal service.

The selected sample consisted of twins aged between 60 and 85 years from throughout Italy. A total of 844 questionnaires were sent out, and 483 were returned, corresponding to a participation rate of approximately 54.5%. This rate was in line with the values observed in all previous ITR surveys. Furthermore, socio-demographic characteristics were similar between participants and non-participants, with the latter subjects including those who either declined participation or did not receive or return the questionnaire. In particular, the distributions of age (mean 69.9 years in participants and 69.2 years in non-participants), sex (63.4% women among participants and 66.3% among non-participants), marital status (married or cohabiting: 70.6% and 67.5% in participants and non-participants, respectively), education (79.7% and 75.0% with an educational level higher than a high school diploma among participants and non-participants, respectively), and area of residence (52.3% of participants and 57.6% of non-participants living in Central Italy) did not differ significantly between the two groups). This provided at least partial reassurance against major socio-demographic selection biases.

Zygosity was determined mainly at the time of enrolment in the ITR, using a validated questionnaire assessing physical similarities and differences within the twin pair during childhood or adolescence. This method has an estimated accuracy of approximately 95% [20].

The study was reviewed and approved by the ISS National Ethics Committee in April 2023 (0017087 Class: PRE BIO CE 01.00). Each participant received informational materials and provided written informed consent before participation in the study.

### 2.2. Measures

Each twin received at home a battery of questionnaires for investigating socio-demographic characteristics, clinical history, and exposure to risk factors including various lifestyle factors, medical conditions, and other potential influences that may either increase or reduce the risk of developing mild cognitive impairment (MCI) or dementia. The checklist-based questionnaire used in this study was the one identified by the World Health Organization [21] and the Lancet Commission [1], which specifically investigates 14 risk factors: (1) lower levels of education, (2) excessive alcohol consumption, (3) hearing loss, (4) high blood pressure, (5) obesity, (6) head injuries, (7) physical inactivity, (8) smoking, (9) vision loss, (10) diabetes, (11) depression, (12) social isolation, (13) unhealthy diet, and (14) exposure to air pollution. Sleep disturbances and COVID-19 were also added based on recent available evidence on their association with incident cognitive decline [22,23]. Age was included as the only non-modifiable risk factor for cognitive impairment

Cognitive abilities were assessed using the Self-Administered Gerocognitive Examination (SAGE) [24,25], a pragmatic first-line tool aimed at flagging potential cognitive concerns rather than detecting preclinical or subtle cognitive changes. It has been previously validated in community-based cohorts and in memory clinics, showing good sensitivity and specificity for the detection of MCI and early dementia [24,25], and performs comparably to the MoCA in screening accuracy [26]. Moreover, SAGE has been effectively used in large-scale mail surveys [27]. These studies support its use in epidemiological and community contexts where neuropsychological assessment is not feasible.

The questionnaires were evaluated and scored by an expert neurologist (NL) and a neuropsychologist (FS). The SAGE score ranges from 0 to 22, with lower scores indicating reduced cognitive performance. A score of 17 or higher is indicative of normal cognitive functioning.

### 2.3. Statistical Analyses

As a first step, a descriptive analysis was conducted, and the mean (SD) of the SAGE score was calculated for all 17 risk factors considered. A correlation matrix of the risk factors was also computed for the entire sample, as well as separately for participants with normal cognitive functioning and those with impaired cognitive functioning (results are presented in the Supplementary Materials). Then, regression models were fitted to estimate the association of risk factors with the SAGE score. Regression analyses were performed using both continuous and dichotomized SAGE score (based on the cut-off of 17). The continuous approach preserves the full variability of the data without loss of information due to categorization, and reduces the risk of using arbitrary cut-offs that may bias results. On the other hand, dichotomized analysis facilitates clinical interpretation and allows direct estimation of risk factors and associations in relation to the presence or absence of cognitive dysfunction. In early-stage regression analyses, twins were treated as separate individuals, while in subsequent analyses, the within-pair matching was considered. More precisely, in the individual-level analysis, the association between each risk factor and cognitive abilities was assessed using the SAGE score both as a continuous variable (via multiple linear regression) and as a categorical variable (via unconditional logistic regression). The strength of the association was assessed considering the estimated beta coefficient and odds ratio. Both models included age, sex, and years of education as covariates. In addition, a pair identifier was included in the models to account for the non-independence of observations, as the sample consisted of twin pairs. As a second step, a matched-pair analysis aimed at comparing the outcome (i.e., SAGE continuous or dichotomized score) between the two twins within a pair (“co-twin control” design) was performed to control for potential confounding due to genetic and environmental factors. This represents a unique approach in classical epidemiology, which allows to account for both observed and unobserved confounding, by taking advantage of the fact that MZ and DZ twins are matched not only for genetics (completely for MZ twins and partially for DZ twins), but also for age, cohort effects, maternal influences, and many shared environmental exposures [14]. The matched analyses were performed considering MZ and DZ twins together, as the number of twin pairs did not allow for reliable zygosity-specific modelling.

When the outcome variable was considered in dichotomous form (i.e., SAGE scores dichotomized as below or above the threshold of 17), a conditional logistic regression analysis was performed, adjusting for sex and years of education. In this analysis, only twin pairs discordant for the outcome—where the normal cognitive twin serves as a matched control for the impaired cognitive co-twin—contributed to the estimated association between the risk factors and cognitive impairment. When the outcome variable was the continuous SAGE score, a fixed-effects regression model was applied, again adjusting for sex and years of education. In this analysis, only complete twin pairs—where twins are compared to matched co-twins in terms of SAGE score—contributed to the estimate of beta coefficients.

These analyses could generate two possible scenarios: (1) the association persists and is equal in magnitude in all analyses (i.e., individual-level and matched-pair analyses), being consistent with a “quasi-causal” effect; (2) the association detected in individual-level analysis weakens or vanishes in matched MZ/DZ analysis, suggesting genetic or shared environmental confounding [14].

All data analyses were conducted using the Stata Statistical Software, Release 16 (StataCorp LLC, College Station, TX, USA).

## 3. Results

A total of 483 twins participated in the study. In some cases, only one twin from a pair responded, resulting in 216 complete twin pairs. The mean age of the participants was 69.14 years, and 63% of them were women. Zygosity was approximately equally distributed between MZ and DZ. The average SAGE score was above 18, with 24% of subjects exceeding the cut-off level of 17 for normal cognitive functioning. Additional characteristics of the sample are presented in Table 1.

No significant sex differences in the SAGE score emerged (male 18.33, female 18.35). Discordant pairs based on the SAGE score (one twin below and the other above the cut-off) represented 29.17% of the sample of complete twin pairs (N = 63 twin pairs).

**Figure 1:**
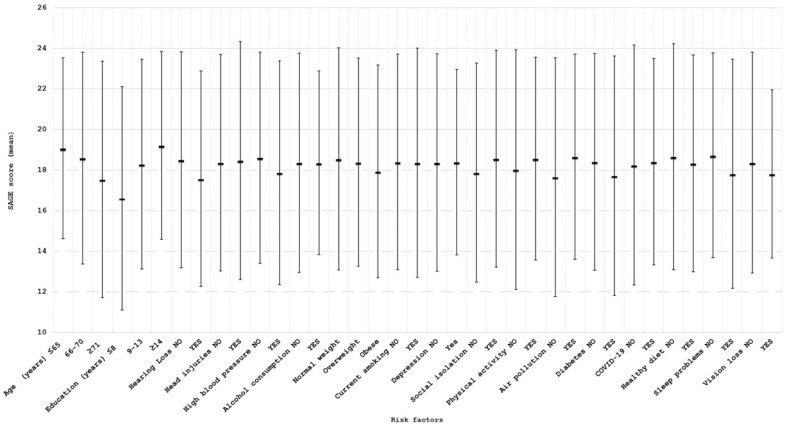
Mean SAGE scores (±2 standard deviations) for all the considered risk factors.

**Figure 2:**
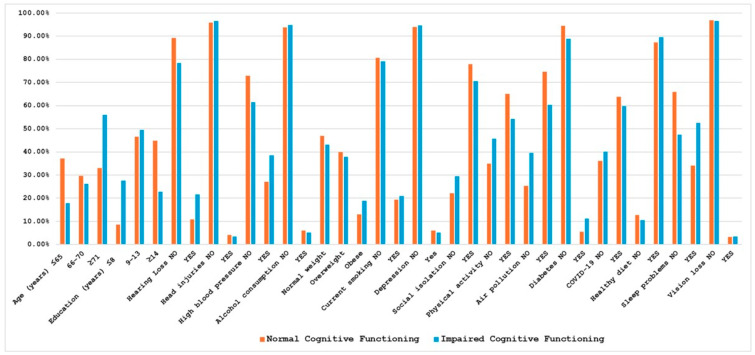
Percentage distribution of risk factors in normal and impaired cognitive subjects. Correlation analyses among all risk factors, conducted for the total sample as well as separately for the groups with normal and impaired cognitive functioning, revealed distinct patterns of associations; the estimated correlation coefficients are provided in the Supplementary Materials (Tables S2–S4).

Figure 1 and Figure 2 show the mean SAGE scores and the prevalence of exposure in subjects with normal or impaired cognitive assessment for the 17 considered risk factors. For clarity and completeness, a table containing all information related to the mean SAGE scores and the percentage distribution of the risk factors has been included in the Supplementary Materials (Table S1). As expected, these scores tended to decrease for older ages and to increase for higher education levels; furthermore, individuals with cognitive impairment showed a higher prevalence of some of the potential risk factors, including hearing loss (21.62% vs. 10.89%), hypertension (38.60% vs. 27.02%), diabetes (11.21% vs. 5.52%), and sleep problems (52.63% vs. 34.07%).

Table 2 shows the results of individual-level and matched-pair analyses of the association between risk factors and cognitive status.

As regards individual-level analysis, multiple linear and logistic regression models (in which the outcome—either the continuous SAGE score or its dichotomized version—was regressed on each individual risk factor, adjusting for age, sex, and education level) indicate lower cognitive performance or an increased MCI risk among participants with untreated hearing loss (Beta = −0.78, *p* = 0.02; OR = 2.33, *p* < 0.01) or disrupted sleep patterns (Beta = −0.96, *p* < 0.001; OR = 2.41, *p* < 0.01). For the other risk factors, a trend toward lower SAGE scores or odds ratios greater than 1 was observed among those subjects in the risk categories, although these associations did not reach statistical significance (e.g., high blood pressure: Beta = −0.52, OR = 1.5; Obesity: Beta = −0.24, OR = 1.14; Diabetes: Beta = −0.02, OR = 1.48). Regression coefficients could not be estimated for some risk factors due to the limited number of exposed subjects.

Concerning matched-pair analysis restricted to those risk factors that showed a significant individual-level association, for hearing loss, the magnitude of the estimated beta coefficient and odds ratio decreased slightly and lost significance after controlling for shared familial factors (i.e., genetics and early-life environment) (Beta = −0.78 vs. −0.03; OR = 2.33 vs. 1.23), suggesting confounding effects by these factors. In contrast, the association between sleep disturbances and reduced cognitive performance remained stable and significant even after accounting for familial influences (Beta = −0.96 vs. −0.77; OR = 2.41 vs. 2.52), supporting the hypothesis of a real, unconfounded association between sleep quality and cognitive health.

## 4. Discussion

This study stands out for examining the association between dementia risk factors and cognitive impairment in a non-clinical, population-based sample of adult twins from the Italian Twin Registry. Rather than simply identifying risk factors for dementia by using the classical checklist for this condition, our main objective was to investigate the causal relationships among 17 risk factors linked to cognitive impairment, both clinical and subclinical. Many studies assessing the role of various risk factors in dementia onset struggle to control for confounding effects related to genetic and environmental factors [1]. Conversely, in our study, a twin pair-based design (matched analysis) was employed to optimally control for variables such as age, genetic background, and early-life environmental exposures, allowing for a more accurate estimation of the associations, independent of these confounding factors.

Classical twin studies represent a powerful tool for disentangling the relative contributions of genetic and environmental influences on a given outcome, as well as for estimating exposure effects while accounting for both measured and unmeasured confounding factors, such as genetic predisposition and early-life influences. This approach is well-suited for investigating complex traits, including dementia and cognitive decline, which result from an intricate interplay between innate and acquired factors. Moreover, an endless list of studies conducted in several countries have demonstrated that, for most traits of interest in biomedical research, the results of twin studies can be generalized to the broader population.

The novelty of this study also lies in its consideration of all known and emerging risk factors—such as sleep disturbances and COVID-19—associated with the onset of dementia. This paradigm contrasts with many twin studies that typically focused on a single factor at a time [15,16,17,18]. The current analysis demonstrates a consistent association between sleep disturbances and cognitive impairment, indicating a limited influence of confounding variables and supporting a putative quasi-causal relationship between this risk factor and cognitive outcomes. This aligns with evidence from a recent meta-analysis encompassing 35 studies, which identified insomnia (RR = 1.43; 95% CI, 1.17–1.74), sleep-disordered breathing (RR = 1.22; 95% CI, 1.07–1.39), and additional sleep-related disorders—including sleep fragmentation and sleep-related movement disorders (RR = 1.58; 95% CI, 1.21–2.07)—as significant risk factors for Alzheimer’s Disease (AD); excessive daytime sleepiness demonstrated a marginally significant association with AD risk (RR = 1.18; 95% CI, 1.00–1.40) [22]. Two additional reviews, based on 27 studies [28] and 15 studies [29], also found that sleep problems or disorders are associated with elevated risk of cognitive impairment or AD. Moreover, two longitudinal studies, which considered twins from Northern European twin registries, reported an association of several sleep characteristics with dementia and cognitive decline [30,31]. It is important to point out that our study, using a screening tool like SAGE in a general population sample, is not directly comparable to the above mentioned studies that focused on clinical MCI or AD. Despite this difference in outcome definition, we could confirm the important role of sleep quality in shaping cognitive functioning. In addition, as a key novelty of our genetically informed twin-matched design, we found some evidence that the effect of sleep on cognition may be independent of familial confounding factors.

From a pathophysiological perspective, sleep disturbances have been linked with an increase in oxidative stress and the accumulation of Aβ and tau proteins, as well as a decrease in melatonin release and a reduction in glymphatic clearance, ultimately leading to damage to the blood–brain barrier. All these phenomena can contribute to the development of AD [32] and cognitive deterioration.

In contrast, the association between hearing loss and cognitive impairment appears to be subject to confounding. In a meta-analysis of six higher quality studies, hearing loss was found associated with an increased risk of developing dementia (HR, 1.37; 95% CI, 1.00—1.87) [1]; however, the evidence on the causal role is limited, and the link between hearing impairment and dementia may not be direct, but rather mediated by other factors [33].

Overall, our study did not find evidence of an association between cognitive decline and most of the risk factors analyzed. This might have been expected from the fact that our study outcome is not diagnosed and clinically manifest dementia or MCI, but rather levels of cognitive impairment that are often sub-threshold or mild, such as those commonly observed in the general population. Additionally, the relatively small sample size may have limited the capacity to detect subtle differences. In particular, statistical power issues should be kept in mind when interpreting the lack of significant associations in the matched-pair analyses in terms of possible genetic or shared environmental confounding factors affecting these associations. It is noteworthy that the analyses conducted on SAGE scores on both continuous and dichotomous (cut-off-based) scale led to the same conclusions; this should not be taken for granted and places our findings in a broader population context.

Despite this study presents several key strengths, including a representative sample of the general Italian population, its substantial sample size, and the utilization of a validated cognitive screening tool, several limitations should be addressed.

Specific risk factors, such as head injuries, excessive alcohol consumption, depression, and vision loss, were not analyzed due to a low number of exposed individuals. The SAGE questionnaire was self-administered, raising potential concerns regarding completion accuracy. Moreover, the SAGE instrument, while validated and used for community screening, is not designed to capture subtle or preclinical cognitive changes. Therefore, our findings should be interpreted as reflecting general cognitive screening outcomes rather than detailed neuropsychological performance. Notably, the Positive Predictive Value for various SAGE score cut-offs related to MCI or dementia diagnosis is low (below 50%), particularly for the 10% prevalence of cognitive disturbances, which is likely the most accurate base rate for primary care settings [34]. We were unable to conduct separate analyses for MZ and DZ twins, which showed poorly reliable estimates with wide confidence intervals. Besides that, the cross-sectional design limits our ability to determine the direction of associations; while we can identify correlations between sleep disorders and MCI or dementia, we cannot establish causality or infer which factor precedes the other. Furthermore, it is likely that some twins already diagnosed with dementia or MCI did not participate in the study; this raises concerns about the low representativeness of the study sample with respect to the more severe cases of cognitive impairment. Other weaknesses are self-reported rather than objectively measured exposure factors, which could introduce recall bias, as well as residual confounding in the association between sleep and cognition, due to individual environmental factors (e.g., lifestyles, medication use) that remain unmatched even in twin pairs. As regards measurement quality, some previous studies considered objective measures like polysomnography or actigraphy for sleep problem assessment, which we should also incorporate in future investigations to validate our results.

In conclusion, our findings suggest that, among the candidate modifiable risk factors for dementia, sleep disorders may play a key role in the onset of cognitive problems within the general population. These findings should be regarded as preliminary and should be interpreted with caution, in the light of the study weaknesses outlined above; thus, they warrant confirmation in subsequent investigations. In this respect, further research should be based on a longitudinal design and should use precise clinical definitions and objective measures—including biomarker data—of various sleep disorders and different forms of MCI and dementia.

## Figures and Tables

**Table 1:** Characteristics of the sample.

	N	%
**Age classes, years**		
≤65	157	32.51%
66–70	139	28.78%
≥71	187	38.72%
**Age, years (mean ± SD)**	69.14 ± 6.20	
**Sex**		
Female	303	62.73%
**Marital status**		
Single	67	14.02%
Married	286	59.83%
Divorced/Separated	86	17.99%
Widowed	39	8.16%
**Years of education (mean ± SD)**	13.72 ± 4.05	
**Years of education, classes**	59	8.16%
≤8	215	47.25%
9–13	181	39.78%
≥14		
**Zygosity**		
MZ	225	47.37%
**Area of residence**		
Northern Italy	204	42.24%
Central Italy	257	53.21%
Southern Italy	22	4.55%
**SAGE score (mean ± SD)**	18.35 ± 2.69	
**SAGE score below 17**	118	24.43%
**SAGE score below 17 (mean ± SD)**	14.47 ± 1.75	
Discordant pairs for SAGE score (above–below 17) *	63	29.17%

* Calculated over 216 complete twin pairs.

**Table 2:** Beta Coefficients and Odds Ratios from Individual and Matched Analyses.

		Individual-Level Analyses				Matched-Pair Analysis MZ and DZ Twin Pairs			
		OR ^1^	*p*	Beta ^2^	*p*	OR ^3^	*p*	Beta ^4^	*p*
**Hearing loss**		2.327	0.007	−0.779	0.021	1.229	0.684	−0.031	0.950
**Head injuries**		nc		nc		nc		nc	
**High blood pressure**		1.492	0.128	−0.521	0.069	1.314	0.597	−0.407	0.334
**Excessive alcohol consumption**		nc		nc		nc		nc	
**BMI**	Overweight	0.872	0.611	−0.024	0.925	1.420	0.519	−0.166	0.700
	Obese	1.139	0.700	−0.240	0.494	9.134	0.050	−1.221	0.036
**Current smoking**		1.064	0.858	−0.102	0.767	0.628	0.478	0.454	0.397
**Depression**		nc		nc		1.317	0.789	−0.093	0.886
**Social isolation**		0.869	0.651	0.317	0.331	2.187	0.274	−0.258	0.579
**Physical inactivity**		0.785	0.337	0.212	0.409	0.899	0.821	0.108	0.769
**Exposure to air pollution**		0.782	0.348	0.409	0.123	0.463	0.200	0.601	0.183
**Diabetes**		1.483	0.394	−0.016	0.975	4.202	0.209	−0.436	0.567
**COVID infection**		0.911	0.707	−0.043	0.873	1.083	0.872	−0.602	0.123
**Healthy Diet**		1.339	0.457	−0.414	0.275	0.159	0.093	0.643	0.201
**Sleep problems**		2.412	0.001	−0.958	0.000	2.521	0.046	−0.770	0.027
**Vision loss**		nc		nc		nc		nc	

^1^ Odds ratios (OR) were estimated using unconditional logistic regression models adjusted for age, sex and years of education. ^2^ Beta coefficients (Beta) were estimated using multiple linear regression models adjusted for age, sex and years of education. ^3^ Odds ratios (OR) were estimated using conditional logistic regression models adjusted for sex and years of education. ^4^ Beta coefficients (Beta) were estimated using fixed-effects regression models adjusted for sex and years of education. nc: not calculated due to the low number of subjects.

## Data Availability

Data are available from the corresponding author upon reasonable request due to ethical reasons.

## Supplementary Materials

The following supporting information can be downloaded at: https://www.mdpi.com/article/10.3390/brainsci15111197/s1, Table S1: Mean and standard deviation of SAGE scores according to risk factors, and percentage distribution of risk factors in normal versus impaired cognitive subjects; Table S2: Pairwise correlations between all risk factors considered–Total sample. For each pair of factors, the first line displays the correlation coefficient, the second line the *p*-value, the third line the number of observations. Table S3: Pairwise correlations between all risk factors considered-Subjects with Normal Cognitive Functioning. For each pair of factors, the first line displays the correlation coefficient, the second line the *p*-value, the third line the number of observations. Table S4: Pairwise correlations between all risk factors considered-Subjects with Impaired Cognitive Functioning. For each pair of factors, the first line displays the correlation coefficient, the second line the *p*-value, the third line the number of observations.

## Abbreviations

The following abbreviations are used in this manuscript:

RCTs	Randomized Controlled Trials
WHO	World Health Organization
SAGE	Self-Administered Gerocognitive Examination
MCI	Mild Cognitive Impairment
AD	Alzheimer’s Disease
ITR	Italian Twin Registry
ISS	Italian National Institute of Health
MZ	Monozygotic twins
DZ	Dizygotic twins
TBI	Traumatic Brain Injury
RR	Relative Risk
CI	Confidence Interval
OR	Odds Ratio
HR	Hazard Ratio
SD	Standard Deviation
