# Supplementary material for: Assessment of Risk Factors for Cognitive Impairment Accounting for Genetic and Environmental Influences: An Italian Population-Based Twin Study
Source: Brain Sci. 2025 Nov 7;15(11):1197. doi: 10.3390/brainsci15111197 (PMC12650674; doi:10.3390/brainsci15111197)
Supplement: Supplementary file 1 [file brainsci-15-01197-s001.zip › brainsci-3872484-supplementary_revised.pdf]

Table S1. Mean and standard deviation of SAGE scores according to risk factors, and percentage distribution of risk factors in normal versus impaired cognitive subjects.

	**SAGE Scores Total Sample (N = 483)**			**Normal Cognitive Functioning (N = 365)**		**Impaired Cognitive Functioning (N = 118)**		***p***
	**N**	**Mean**	**SD**	**N**	**%**	**N**	**%**	
**Age**								
≤65	157	19.077	2.228	136	37.26%	21	17.80%	
66–70	139	18.594	2.610	108	29.59%	31	26.27%	
≥71	187	17.535	2.915	121	33.15%	66	55.93%	<0.001
**Education (years)**								
≤8	59	16.610	2.754	30	8.57%	29	27.62%	
9–13	215	18.293	2.580	163	46.57%	52	49.52%	
≥14	181	19.215	2.313	157	44.86%	24	22.86%	<0.001
**Hearing loss**								
No	406	18.505	2.660	319	89.11%	87	78.38%	
Yes	63	17.571	2.656	39	10.89%	24	21.62%	0.004
**Head injuries**								
No	458	18.365	2.665	347	95.86%	111	96.52%	
Yes	19	18.474	2.932	15	4.14%	4	3.48%	0.75
**High blood pressure**								
No	332	18.608	2.603	262	72.98%	70	61.40%	
Yes	141	17.872	2.756	97	27.02%	44	38.60%	0.02
**Excessive alcohol consumption**								
No	446	18.363	2.703	337	93.87%	109	94.78%	
Yes	28	18.357	2.264	22	6.13%	6	5.22%	0.72
**BMI**								
Normal weight	216	18.551	2.734	168	46.93%	48	43.24%	
Overweight	185	18.389	2.564	143	39.94%	42	37.84%	
Obese	68	17.941	2.620	47	13.13%	21	18.92%	0.32
**Current smoking**								
No	370	18.405	2.658	283	80.63%	87	79.09%	
Yes	91	18.363	2.823	68	19.37%	23	20.91%	0.72
**Depression**								
No	446	18.374	2.679	338	93.89%	108	94.74%	
Yes	28	18.393	2.283	22	6.11%	6	5.26%	0.74
**Social isolation**								
No	112	17.875	2.698	79	22.13%	33	29.46%	
Yes	357	18.563	2.668	278	77.87%	79	70.54%	0.11
**Physical activity**								
No	179	18.022	2.955	126	34.90%	53	45.69%	
Yes	298	18.567	2.495	235	65.10%	63	54.31%	0.04
**Exposure to air pollution**								
No	138	17.652	2.941	92	25.41%	46	39.66%	
Yes	340	18.656	2.529	270	74.59%	70	60.34%	0.003
**Diabetes**								
No	445	18.409	2.673	342	94.48%	103	88.79%	
Yes	33	17.727	2.950	20	5.52%	13	11.21%	0.04
**COVID-19**								
No	178	18.247	2.956	131	36.19%	47	40.17%	
Yes	301	18.409	2.543	231	63.81%	70	59.83%	0.44
**Healthy diet**								
No	58	18.655	2.782	46	12.74%	12	10.53%	
Yes	417	18.331	2.673	315	87.26%	102	89.47%	0.53
**Sleep problems**								
No	292	18.729	2.527	238	65.93%	54	47.37%	
Yes	183	17.820	2.822	123	34.07%	60	52.63%	<0.001
**Vision loss**								
No	463	18.367	2.721	350	96.96%	113	96.58%	
Yes	16	17.813	2.073	12	3.31%	4	3.42%	0.96

p-values: Chi-square test

Table S2: Pairwise correlations between all risk factors considered – Total sample.
For each pair of factors, the first line displays the correlation coefficient, the second line the p-value, the third line the number of observations.

	Age	Sex	Education	Hearing L.	Head I.	High B.	Alcohol C.	BMI	Smoke	Depression	Social I.	Physical A.	Pollution	Diabetes	Covid	H.Diet	Sleep P.	Vision L.
**Age, classes**	**1.0000**																	
	483																	
**Sex**	**-0.0445**	**1.0000**																
	0.3327																	
	476	*476*																
**Education**	**-0.1647**	**-0.1560**	**1.0000**															
	0.0004	0.0009																
	455	*452*	*455*															
**Hearing loss**	**0.1249**	**-0.0544**	**-0.0569**	**1.0000**														
	0.0068	0.2408	0.2301															
	469	*466*	*447*	*469*														
**Head injuries**	**-0.0536**	**0.0039**	**0.0306**	**0.0837**	**1.0000**													
	0.2429	0.9328	0.5164	0.0706														
	477	*474*	*453*	*467*	*477*													
**High blood Pressure**	**0.1814**	**-0.1367**	**-0.0138**	**0.0993**	**-0.0019**	**1.0000**												
	0.0001	0.0030	0.7708	0.0327	0.9663													
	473	*470*	*450*	*463*	*472*	*473*												
**Excessive alcohol consumption**	**-0.0076**	**-0.1374**	**-0.0150**	**0.0069**	**-0.0031**	**0.1786**	**1.0000**											
	0.8691	0.0028	0.7515	0.8825	0.9469	0.0001												
	474	*471*	*450*	*464*	*473*	*468*	*474*											
**BMI, classes**	**0.0566**	**-0.2029**	**-0.1054**	**0.1494**	**0.0606**	**0.1921**	**0.0610**	**1.0000**										
	0.2215	0.0000	0.0260	0.0013	0.1911	0.0000	0.1894											
	469	*466*	*446*	*459*	*467*	*463*	*464*	*469*										
**Current smoke**	**-0.0605**	**-0.0107**	**0.0293**	**0.0567**	**0.0410**	**0.0174**	**0.1311**	**-0.0312**	**1.0000**									
	0.1946	0.8207	0.5422	0.2313	0.3829	0.7116	0.0052	0.5096										
	461	*455*	*436*	*448*	*456*	*454*	*453*	*448*	*461*									
**Depression**	**-0.0384**	**0.1353**	**0.0182**	**0.0433**	**0.0886**	**0.0039**	**0.0566**	**0.0009**	**-0.0034**	**1.0000**								
	0.4048	0.0034	0.7009	0.3538	0.0549	0.9339	0.2229	0.9853	0.9424									
	474	*468*	*449*	*461*	*470*	*466*	*466*	*461*	*455*	*474*								
**Social isolation**	**0.0103**	**0.0505**	**0.1560**	**0.0214**	**-0.0955**	**-0.0662**	**0.0117**	**-0.0903**	**0.0131**	**0.0318**	**1.0000**							
	0.8243	0.2771	0.0009	0.6483	0.0395	0.1553	0.8012	0.0534	0.7811	0.4949								
	469	*465*	*446*	*458*	*465*	*462*	*462*	*458*	*450*	*463*	*469*							
**Physical Activity**	**-0.0644**	**-0.0823**	**0.0728**	**-0.1156**	**0.0021**	**-0.1125**	**-0.1028**	**-0.1246**	**-0.0612**	**-0.0738**	**0.0814**	**1.0000**						
	0.1601	0.0742	0.1221	0.0125	0.9634	0.0148	0.0257	0.0071	0.1911	0.1095	0.0785							
	477	*472*	*452*	*466*	*474*	*469*	*471*	*465*	*458*	*471*	*468*	*477*						
**Air Pollution**	**-0.0599**	**-0.0143**	**0.2270**	**-0.0019**	**0.0586**	**-0.0564**	**0.0417**	**-0.0145**	**0.0383**	**0.0816**	**0.1715**	**0.0095**	**1.0000**					
	0.1911	0.7564	0.0000	0.9681	0.2034	0.2220	0.3674	0.7555	0.4134	0.0767	0.0002	0.8359						
	478	*472*	*452*	*465*	*473*	*470*	*470*	*465*	*459*	*472*	*467*	*475*	*478*					
**Diabetes**	**0.1487**	**-0.1439**	**-0.0695**	**0.0663**	**-0.0122**	**0.0703**	**0.0747**	**0.1695**	**-0.0454**	**-0.0319**	**-0.1209**	**0.0234**	**0.0039**	**1.0000**				
	0.0011	0.0017	0.1397	0.1537	0.7907	0.1287	0.1057	0.0002	0.3313	0.4889	0.0089	0.6114	0.9325					
	478	*472*	*453*	*465*	*473*	*469*	*470*	*465*	*459*	*473*	*467*	*475*	*476*	*478*				
**Covid-19**	**-0.0198**	**0.0160**	**0.0592**	**-0.0557**	**0.0003**	**0.0115**	**-0.0866**	**-0.0455**	**-0.0517**	**-0.0130**	**0.0517**	**0.0197**	**0.0622**	**-0.0655**	**1.0000**			
	0.6655	0.7283	0.2084	0.2301	0.9943	0.8030	0.0603	0.3272	0.2685	0.7779	0.2643	0.6689	0.1753	0.1534				
	479	*473*	*453*	*466*	*474*	*470*	*471*	*466*	*460*	*473*	*468*	*476*	*477*	*477*	*479*			
**Healthy diet**	**0.0654**	**0.0973**	**-0.0434**	**0.0102**	**-0.0579**	**-0.0336**	**-0.1036**	**-0.0521**	**0.0042**	**-0.0147**	**0.1445**	**0.1310**	**-0.0217**	**-0.1559**	**-0.0006**	**1.0000**		
	0.1549	0.0351	0.3594	0.8265	0.2103	0.4686	0.0252	0.2640	0.9293	0.7510	0.0018	0.0043	0.6380	0.0007	0.9897			
	475	*469*	*449*	*462*	*470*	*467*	*467*	*462*	*456*	*469*	*465*	*472*	*474*	*473*	*474*	*475*		
**Sleep problems**	**-0.0144**	**0.0368**	**-0.0865**	**0.0573**	**0.0605**	**0.1243**	**0.1694**	**0.0627**	**0.0052**	**0.0040**	**-0.1091**	**-0.1126**	**0.1102**	**0.0296**	**0.0478**	**-0.0932**	**1.0000**	
	0.7547	0.4266	0.0670	0.2191	0.1903	0.0072	0.0002	0.1786	0.9115	0.9309	0.0188	0.0144	0.0164	0.5201	0.2990	0.0431		
	475	*469*	*449*	*462*	*470*	*467*	*467*	*462*	*456*	*470*	*464*	*472*	*474*	*474*	*474*	*471*	*475*	
**Vision loss**	**-0.0824**	**0.0013**	**-0.0929**	**0.0302**	**0.0809**	**0.0396**	**0.0520**	**0.0493**	**0.0010**	**0.0057**	**-0.0685**	**0.0485**	**-0.0866**	**0.0869**	**-0.0732**	**-0.1084**	**0.1637**	**1.0000**
	0.0716	0.9771	0.0483	0.5150	0.0784	0.3914	0.2602	0.2878	0.9829	0.9011	0.1388	0.2907	0.0589	0.0580	0.1100	0.0182	0.0003	
	479	*473*	*453*	*466*	*474*	*470*	*471*	*466*	*460*	*473*	*468*	*476*	*477*	*477*	*478*	*474*	*474*	*479*

Table S3: Pairwise correlations between all risk factors considered - Subjects with Normal Cognitive Functioning.
For each pair of factors, the first line displays the correlation coefficient, the second line the p-value, the third line the number of observations.

	Age	Sex	Education	Hearing L.	Head I.	High B.	Alcohol C.	BMI	Smoke	Depression	Social I.	Physical A.	Pollution	Diabetes	Covid	H.Diet	Sleep P.	Vision L.
**Age, classes**	1.0000																	
	365																	
**Sex**	-0.0493	1.0000																
	0.3497																	
	362	362																
**Education**	-0.1015	-0.1527	1.0000															
	0.0578	0.0043																
	350	348	350															
**Hearing loss**	0.0829	-0.0452	0.0199	1.0000														
	0.1176	0.3955	0.7127															
	358	356	345	358														
**Head injuries**	-0.0235	-0.0108	0.0309	0.0678	1.0000													
	0.6556	0.8387	0.5662	0.2016														
	362	360	348	356	362													
**High blood**	0.1374	-0.1340	0.0632	0.0217	-0.0175	1.0000												
**Pressure**	0.0092	0.0113	0.2407	0.6840	0.7407													
	359	357	346	353	358	359												
**Excessive**	-0.0150	-0.1141	-0.0344	-0.0139	0.0084	0.1965	1.0000											
**alcohol**	0.7774	0.0312	0.5238	0.7943	0.8745	0.0002												
**consumption**	359	357	345	353	358	354	359											
**BMI**	0.0523	-0.2227	-0.0435	0.1057	0.0612	0.1829	0.0572	1.0000										
	0.3238	0.0000	0.4218	0.0474	0.2494	0.0006	0.2838											
	358	356	344	352	356	353	353	358										
**Current smoke**	-0.0473	-0.0238	0.0297	0.0956	0.0466	0.0355	0.1508	-0.0153	1.0000									
	0.3765	0.6578	0.5871	0.0765	0.3858	0.5098	0.0050	0.7775										
	351	348	337	344	348	347	345	344	351									
**Depression**	-0.0020	0.1265	-0.0123	0.0672	0.1206	0.0355	0.0344	-0.0168	0.0029	1.0000								
	0.9698	0.0168	0.8195	0.2078	0.0224	0.5048	0.5186	0.7533	0.9576									
	360	357	346	353	358	355	354	353	348	360								
**Social**	0.0278	0.0820	0.2046	0.0074	-0.0653	-0.0274	0.0209	-0.0766	0.0172	0.0239	1.0000							
**isolation**	0.6003	0.1234	0.0001	0.8894	0.2204	0.6085	0.6964	0.1526	0.7499	0.6537								
	357	354	344	351	354	352	351	350	345	354	357							
**Physical**	-0.0294	-0.0783	0.0375	-0.0351	0.0065	-0.0621	-0.1062	-0.0961	-0.0248	-0.0810	0.0274	1.0000						
**Activity**	0.5772	0.1392	0.4858	0.5096	0.9021	0.2433	0.0453	0.0710	0.6441	0.1262	0.6058							
	361	358	347	355	359	355	356	354	349	358	356	361						
**Air Pollution**	-0.0364	0.0201	0.1872	0.0971	0.0577	0.0076	0.0434	-0.0016	0.0712	0.0968	0.1830	-0.0294	1.0000					
	0.4897	0.7036	0.0004	0.0678	0.2758	0.8866	0.4141	0.9765	0.1841	0.0670	0.0005	0.5785						
	362	359	348	355	359	356	356	355	350	359	356	360	362					
**Diabetes**	0.1429	-0.1858	-0.0569	0.0766	-0.0494	0.0247	0.0429	0.1716	0.0098	-0.0604	-0.1362	0.0254	0.0012	1.0000				
	0.0065	0.0004	0.2890	0.1499	0.3510	0.6426	0.4201	0.0012	0.8545	0.2537	0.0101	0.6307	0.9825					
	362	359	349	355	359	356	356	355	350	359	356	360	361	362				
**Covid-19**	-0.0005	0.0298	0.0775	-0.0457	0.0113	-0.0016	-0.0994	-0.0191	-0.0892	-0.0008	0.0701	0.0226	0.0742	-0.0958	1.0000			
	0.9931	0.5730	0.1491	0.3904	0.8308	0.9756	0.0609	0.7193	0.0957	0.9878	0.1868	0.6690	0.1593	0.0691				
	362	359	348	355	359	356	356	355	350	359	356	360	361	361	362			
**Healthy diet**	0.0914	0.1122	-0.0242	0.0255	-0.0048	-0.0324	-0.1161	-0.0590	-0.0050	-0.0408	0.1626	0.1295	-0.0503	-0.1251	0.0504	1.0000		
	0.0830	0.0338	0.6536	0.6332	0.9276	0.5431	0.0288	0.2681	0.9257	0.4418	0.0021	0.0141	0.3412	0.0175	0.3407			
	361	358	347	354	358	355	355	354	349	358	355	359	360	360	360	361		
**Sleep problems**	-0.0482	0.0775	-0.0561	0.0237	0.0569	0.1231	0.2322	0.0399	0.0247	-0.0367	-0.0988	-0.0850	0.0551	0.0057	0.0579	-0.1191	1.0000	
	0.3616	0.1436	0.2975	0.6570	0.2831	0.0203	0.0000	0.4546	0.6462	0.4883	0.0630	0.1080	0.2968	0.9143	0.2730	0.0240		
	361	358	347	354	358	355	355	354	349	358	355	359	360	360	360	359	361	
**Vision loss**	-0.1013	0.0164	-0.0822	0.0361	0.1161	0.0366	0.0813	0.0221	0.0357	0.0171	-0.0609	0.0389	-0.1043	0.0226	-0.0529	-0.1143	0.1925	1.0000
	0.0542	0.7568	0.1261	0.4981	0.0279	0.4917	0.1255	0.6783	0.5054	0.7468	0.2517	0.4615	0.0476	0.6681	0.3164	0.0301	0.0002	
	362	359	348	355	359	356	356	355	350	359	356	360	361	361	361	360	360	362

Table S4: Pairwise correlations between all risk factors considered - Subjects with Impaired Cognitive Functioning.
For each pair of factors, the first line displays the correlation coefficient, the second line the p-value, the third line the number of observations.

	**Age**	**Sex**	**Education**	**Hearing L.**	**Head I.**	**High B.**	**Alcohol C.**	**BMI**	**Smoke**	**Depression**	**Social I.**	**PhysicalA.**	**Pollution**	**Diabetes**	**Covid**	**H.Diet**	**Sleep P.**	**Vision L.**
**Age, classes**	**1.0000**																	
	118																	
**Sex**	**-0.0239**	**1.0000**																
	0.8006																	
	114	*114*																
**Education**	**-0.1791**	**-0.2111**	**1.0000**															
	0.0676	0.0314																
	105	*104*	*105*															
**Hearing loss**	**0.1524**	**-0.0730**	**-0.0907**	**1.0000**														
	0.1104	0.4485	0.3644															
	111	*110*	*102*	*111*														
**Head injuries**	**-0.1571**	**0.0533**	**0.0115**	**0.1333**	**1.0000**													
	0.0935	0.5736	0.9071	0.1631														
	115	*114*	*105*	*111*	*115*													
**High blood Pressure**	**0.2449**	**-0.1439**	**-0.1015**	**0.2301**	**0.0447**	**1.0000**												
	0.0086	0.1283	0.3054	0.0156	0.6370													
	114	*113*	*104*	*110*	*114*	*114*												
**Excessive alcohol consumption**	**0.0360**	**-0.2166**	**0.0150**	**0.0680**	**-0.0445**	**0.1359**	**1.0000**											
	0.7022	0.0206	0.8791	0.4782	0.6365	0.1494												
	115	*114*	*105*	*111*	*115*	*114*	*115*											
**BMI**	**0.0270**	**-0.1431**	**-0.2241**	**0.2367**	**0.0627**	**0.1978**	**0.0775**	**1.0000**										
	0.7782	0.1357	0.0236	0.0141	0.5132	0.0383	0.4186											
	111	*110*	*102*	*107*	*111*	*110*	*111*	*111*										
**Current smoke**	**-0.1246**	**0.0320**	**0.0199**	**-0.0372**	**0.0225**	**-0.0393**	**0.0544**	**-0.0804**	**1.0000**									
	0.1948	0.7436	0.8448	0.7079	0.8169	0.6878	0.5760	0.4174										
	110	*107*	*99*	*104*	*108*	*107*	*108*	*104*	*110*									
**Depression**	**-0.1603**	**0.1694**	**0.0855**	**-0.0070**	**-0.0416**	**-0.0872**	**0.1406**	**0.0677**	**-0.0235**	**1.0000**								
	0.0884	0.0754	0.3906	0.9429	0.6632	0.3628	0.1393	0.4860	0.8102									
	114	*111*	*103*	*108*	*112*	*111*	*112*	*108*	*107*	*114*								
**Social Isolation**	**0.0198**	**-0.0436**	**-0.0262**	**0.0894**	**-0.1915**	**-0.1432**	**-0.0188**	**-0.1195**	**0.0040**	**0.0490**	**1.0000**							
	0.8359	0.6498	0.7935	0.3599	0.0441	0.1356	0.8443	0.2179	0.9678	0.6126								
	112	*111*	*102*	*107*	*111*	*110*	*111*	*108*	*105*	*109*	*112*							
**Physical activity**	**-0.0976**	**-0.0973**	**0.1016**	**-0.2623**	**-0.0182**	**-0.2145**	**-0.1011**	**-0.1916**	**-0.1655**	**-0.0643**	**0.2075**	**1.0000**						
	0.2971	0.3032	0.3022	0.0054	0.8466	0.0219	0.2823	0.0440	0.0855	0.4988	0.0281							
	116	*114*	*105*	*111*	*115*	*114*	*115*	*111*	*109*	*113*	*112*	*116*						
**Air Pollution**	**-0.0217**	**-0.1166**	**0.2466**	**-0.1528**	**0.0565**	**-0.1707**	**0.0296**	**-0.0231**	**-0.0426**	**0.0314**	**0.1186**	**0.0641**	**1.0000**					
	0.8175	0.2187	0.0116	0.1111	0.5507	0.0693	0.7545	0.8104	0.6598	0.7413	0.2151	0.4962						
	116	*113*	*104*	*110*	*114*	*114*	*114*	*110*	*109*	*113*	*111*	*115*	*116*					
**Diabetes**	**0.1127**	**-0.0534**	**-0.0230**	**0.0112**	**0.0816**	**0.1371**	**0.1626**	**0.1529**	**-0.1769**	**0.0390**	**-0.0696**	**0.0485**	**0.0464**	**1.0000**				
	0.2285	0.5746	0.8171	0.9079	0.3883	0.1476	0.0839	0.1107	0.0658	0.6801	0.4681	0.6070	0.6221					
	116	*113*	*104*	*110*	*114*	*113*	*114*	*110*	*109*	*114*	*111*	*115*	*115*	*116*				
**Covid-19**	**-0.0526**	**-0.0277**	**-0.0274**	**-0.0665**	**-0.0387**	**0.0644**	**-0.0479**	**-0.1155**	**0.0641**	**-0.0552**	**-0.0103**	**-0.0006**	**0.0130**	**0.0087**	**1.0000**			
	0.5732	0.7698	0.7812	0.4880	0.6810	0.4958	0.6113	0.2274	0.5061	0.5596	0.9138	0.9948	0.8899	0.9264				
	117	*114*	*105*	*111*	*115*	*114*	*115*	*111*	*110*	*114*	*112*	*116*	*116*	*116*	*117*			
**Healthy diet**	**-0.0550**	**0.0457**	**-0.0696**	**-0.0492**	**-0.2598**	**-0.0542**	**-0.0547**	**-0.0354**	**0.0342**	**0.0832**	**0.0969**	**0.1492**	**0.0803**	**-0.2541**	**-0.1656**	**1.0000**		
	0.5608	0.6337	0.4867	0.6134	0.0057	0.5701	0.5666	0.7164	0.7262	0.3852	0.3140	0.1148	0.3955	0.0066	0.0783			
	114	*111*	*102*	*108*	*112*	*112*	*112*	*108*	*107*	*111*	*110*	*113*	*114*	*113*	*114*	*114*		
**Sleep problems**	**-0.0604**	**-0.0741**	**-0.0071**	**0.0595**	**0.0860**	**0.0667**	**-0.0128**	**0.0907**	**-0.0605**	**0.1461**	**-0.1021**	**-0.1368**	**0.3481**	**0.0392**	**0.0433**	**-0.0331**	**1.0000**	
	0.5232	0.4397	0.9434	0.5409	0.3671	0.4848	0.8937	0.3503	0.5356	0.1243	0.2909	0.1484	0.0001	0.6791	0.6470	0.7292		
	114	*111*	*102*	*108*	*112*	*112*	*112*	*108*	*107*	*112*	*109*	*113*	*114*	*114*	*114*	*112*	*114*	
**Vision loss**	**-0.0308**	**-0.0447**	**-0.1270**	**0.0159**	**-0.0360**	**0.0447**	**-0.0445**	**0.1272**	**-0.0999**	**-0.0387**	**-0.0867**	**0.0785**	**-0.0400**	**0.2324**	**-0.1337**	**-0.0899**	**0.0854**	**1.0000**
	0.7418	0.6370	0.1967	0.8687	0.7022	0.6370	0.6365	0.1835	0.2992	0.6823	0.3634	0.4023	0.6702	0.0121	0.1508	0.3413	0.3661	
	117	*114*	*105*	*111*	*115*	*114*	*115*	*111*	*110*	*114*	*112*	*116*	*116*	*116*	*117*	*114*	*114*	*117*
